# *NT5E*/CD73 as Correlative Factor of Patient Survival and Natural Killer Cell Infiltration in Glioblastoma

**DOI:** 10.3390/jcm8101526

**Published:** 2019-09-23

**Authors:** Jiao Wang, Sandro Matosevic

**Affiliations:** 1 Department of Industrial and Physical Pharmacy, Purdue University, West Lafayette, IN 47907, USA; wang3742@purdue.edu; 2 Purdue Center for Cancer Research, West Lafayette, IN 47906, USA

**Keywords:** adenosine, natural killer cells, CD73, glioblastoma, immunometabolism

## Abstract

CD73, a cell-surface protein encoded by the gene *NT5E*, is overexpressed in glioblastoma (GBM), where it contributes to the tumor’s pathophysiology via the generation of immunosuppressive adenosine. Adenosinergic signaling, in turn, drives immunosuppression of natural killer (NK) cells through metabolic and functional reprogramming. The correlation of CD73 with patient survival in relation to GBM pathology and the intratumoral infiltration of NK cells has not been comprehensively studied before. Here, we present an analysis of the prognostic relevance of CD73 in GBM based on transcriptional gene expression from patient data from The Cancer Genome Atlas (TCGA) database. Utilizing bioinformatics data mining tools, we explore the relationship between GBM prognosis, *NT5E* expression, and intratumoral presence of NK cells. Our analysis demonstrates that CD73 is a negative prognostic factor for GBM and that presence of NK cells may associate with improved prognosis. Moreover, the interplay between expression of *NT5E* and specific NK genes hints to potential functional effects of CD73 on NK cell activation.

## 1. Introduction

Glioblastoma multiforme (GBM) is the most aggressive brain tumor, with a median overall survival (OS) of 12–15 months despite aggressive treatment centered around resection based on magnetic resonance imaging analysis followed by radiotherapy and chemotherapy with temozolomide [1]. GBM is characterized by high molecular and cellular heterogeneity within a single tumor and among different patients, which collectively contribute to its pathogenesis and, as a result, its poor response to treatment [2]. GBM invasiveness is favored by hypoxic foci which form in response to defective and leaky tumor vasculature [3,4]. Low tumor oxygenation, or hypoxia, ultimately results in the spreading of GBM to healthy tissues [5]. Hypoxia in GBM is, moreover, heterogeneous, and is often accompanied by abnormal angiogenesis [6], acidosis, necrosis and intratumoral edema [7].

Immunosuppressive adenosine signaling in GBM is one of the consequences of tumor hypoxia. Hypoxia is a potent inducer of the expression of the cancer-associated enzyme ecto-5′-nucleotidase (*NT5E*/CD73). CD73 has been linked to multiple elements of glioblastoma pathogenesis, including growth, angiogenesis, and invasiveness. In tandem with the surface-expressing enzyme ectonucleoside triphosphate diphosphohydrolase–1 (*ENTPD1*/CD39), CD73 produces adenosine from AMP, itself generated by CD39 from pro-inflammatory ATP, which is released from stressed or damaged cells into the extracellular space. Adenosine concentration in the extracellular fluid of glioma tissues was reported to be in the low micromolar range [8], sufficient, however, to stimulate signaling on all four adenosine receptors. These receptors—A_1_, A_2A_, A_2B_, and A_3_—are present on immune cells including natural killer (NK) cells and have been associated with involvement in various immunosuppressive signaling mechanisms [9,10]. 

Though NK cells were detected in intracranial tumors including GBM, their infiltration was dependent on nature of the cancer, origin of metastasis, and stage of disease [11]. Conflicting reports have contributed to our limited knowledge on the presence and exact role of NK cells in GBM [11]. Contrary to reports indicating NK cells to be the least numerous immune population in brain cancer [12], contrasting data have argued that NK cell presence could be substantial in GBM [13]. While the immunosuppressive role of CD73-mediated adenosinergic signaling on NK cells has been described [14,15], the prognostic role of CD73 on GBM and its pathogenesis remains largely underexplored. Moreover, the relationship between expression of and signaling via CD73 and intratumoral infiltration of NK cells is unknown as few studies have explored the association between the expression of CD73 and GBM.

Here, we present an investigation into the expression of CD73 in GBM based on in silico analyses of patient datasets from The Cancer Genome Atlas (TCGA), the Gene Expression Omnibus (GEO), and the Genotype Tissue Expression (GTEx) project. We use Kaplan–Meier survival plots and expression correlation analysis to infer the potential prognostic significance of the expression profiles of CD73 in GBM. We further analyzed the expression of NK cell gene signatures to infer the role of NK cell infiltration in GBM. The aim of this study is to survey publicly-available clinical datasets to infer, using in silico approaches, the significance of CD73 in GBM and any correlation between the presence of NK cells and expression of CD73 and, ultimately, patient outcomes in GBM. Analyses of data revealed that there was selective upregulation of CD73 expression in GBM when compared with normal brain tissue and this correlated to both a poorer survival in distinct GBM subsets and a potentially impaired intratumoral trafficking of NK cells.

## 2. Experimental Section

### 2.1. Transcriptional Data from Clinical Samples

We obtained RNA sequencing (RNASeq) clinical data for glioblastoma multiforme from The Cancer Genome Atlas (TCGA). Log_2_ transformed data were extracted from RNASeq V2 RSEM and Affymetrix U133 array databases. Data from the Gene Expression Omnibus (GEO) were extracted from GSE53733 and GSE36245, obtained using Affymetrix Human Genome U133 Plus 2.0 Arrays. GSE53733 represents gene expression profiling of primary tumor samples from 70 glioblastoma patients of the German Glioma Network, including 23 long-term survivors with >36 months OS, 16 short-term survivors with <12 months OS, and 31 patients with intermediate OS. GSE36245 represents gene expression data for 46 human glioblastoma samples from patients of various ages whose tumors were analyzed from different regions of the brain. Non-disease brain gene expression data were obtained from the Genotype-Tissue Expression (GTEx) project [16]. GTEx catalogues gene expression data across normal brain tissue from healthy individuals. GTEx data were computed using the USC Xena Database by GEPIA2 and utilized for each analysis using this tool. TCGA normal data represent matched tissue normal samples from individuals with GBM.

### 2.2. Tissue-Wide Gene Expression and Correlation Analysis

Tissue-wide analysis of gene expression of individual genes as well as gene signatures was done with GEPIA2, TCGA Wanderer, SurvExpress and R2. GEPIA2 (http://gepia2.cancer-pku.cn/) is a web server for RNAseq expression profiling [17]. GEPIA2 performs data mining based on TCGA data. The log_2_FC cutoff was set as 1, and the *p*-value was set to 0.01. Target gene expression data were compiled based on comparison of GBM TCGA sample data and combined normal data from TCGA and GTEx. SurvExpress (http://bioinformatica.mty.itesm.mx/SurvExpress) is a cancer-wide gene expression database with clinical outcomes and a web-based tool for survival analysis [18]. SurvExpress was used, with TCGA data for glioblastoma multiforme (538 samples), to estimate gene expression levels stratified by risk group. High and low risk groups are defined based on risk prognosis, with higher values indicating higher risk. The prognostic index defining risk groups is estimated by SurvExpress based on the classical Cox model, from which risk groups are determined based on a log-rank test [18]. TCGA Wanderer (http://maplab.imppc.org/wanderer/) was used to perform data mining on level 3 TCGA data to extract gene expression signatures in GBM and normal tissue [19]. R2 (https://hgserver1.amc.nl/) was used to profile gene expression analysis stratified by vital status, based on TCGA U133 microarray data (540 samples, log_2_ transformed).

Correlation of gene expression signatures was determined in GEPIA2, cBioPortal for Cancer Genomics and UALCAN. RNAseq (V2, RSEM) data for select genes were used to determine correlation in gene expression pairs in GEPIA2. For each queried gene pair, Pearson’s and Spearman’s correlation coefficients were determined. Clinical attributes and genomic alterations in the GBM patient cohorts was also visualized. Gene expression analysis for individually-queried genes based on GBM subtype (classical, mesenchymal, neural, and proneural) was generated in GEPIA2. cBioPortal (http://www.cbioportal.org) is an open-access tool developed at the Memorial Sloan-Kettering Cancer Center for analysis of large scale genomics cancer data sets [20]. TCGA U133 microarray co-expression data for selected gene pairs were analyzed based on a z-score threshold of 1, with Spearman’s and Pearson’s correlation coefficients determined for each queried gene pair. UALCAN, a tool for visualization of genomics data, was used to determine positively correlated genes expressed alongside *NT5E* [21]. GEO data were analyzed using shinyGEO (https://gdancik.shinyapps.io/shinyGEO/) [22].

### 2.3. Survival Analysis Based on Gene Expression Data

Survival analysis based on expression of individual queried genes or gene signatures was done in cBioPortal, SurvExpress, and GEPIA2 using TCGA gene expression data. Data were analyzed and generated using a Kaplan–Meier curve for overall (OS) and disease-free survival. Kaplan–Meier curves were generated with a median survival cutoff. The estimation of hazard ratios was done by Cox proportional hazards model regression analysis. A 95% confidence interval was set and used. Survival analysis between individual gene expression data and gene signatures corresponding to infiltrating NK cells was done to determine correlations between survival and immune subsets. The expression threshold for splitting the high-expression and low-expression cohorts was set at 50%. Patient samples with expression level above the threshold were considered as the high-expression or high-risk cohort. A z-value threshold was set to 2 in cBioportal for RNASeq (V2, RSEM) data and 1 for U133 Affymetrix microarray data.

### 2.4. Determination of Tumor-Infiltrating Natural Killer Cells

Level 3 TCGA RNAseq data, mapped to the human genome and collected from the Affymetrix HT Human Genome U133a microarray platform, were extracted. Deconvolution into leukocyte gene signatures was done in bioinformatics tool CIBERSORT (http://sibersort.stanford.edu), an algorithm for the deconvolution of complex cell populations. Individual gene identifiers were adjusted to appropriate HUGO names. Immune cells were identified on the basis of the LM22 signature matrix. Deconvoluted data were manually filtered out based on a *p* < 0.05 statistical cutoff and plotted using a stacked bar graph. Percentages of NK cells in relation to other immune subtypes were determined based on this data (*p* < 0.05).

### 2.5. Statistical Analysis

Expression data were extracted from TCGA, cBioPortal, GEPIA, R2, UALCAN, Xena, and SurvExpress databases. The *p*-values < 0.05 were considered significant (we used * *p* < 0.05 or *p* < 0.01). Survival curves were extracted from the R2, GEPIA2, cBioPortal, and SurvExpress databases. All survival results are displayed with *p*-values obtained using the log-rank test. Immune cell infiltration percentages were deconvoluted in CIBERSORT based on TCGA gene expression data and processed to generate graphs with log-rank *p*-values < 0.05 considered significant.

## 3. Results

### 3.1. Expression of CD73, HIF1A and ENTPD1 in GBM

**Figure 1:**
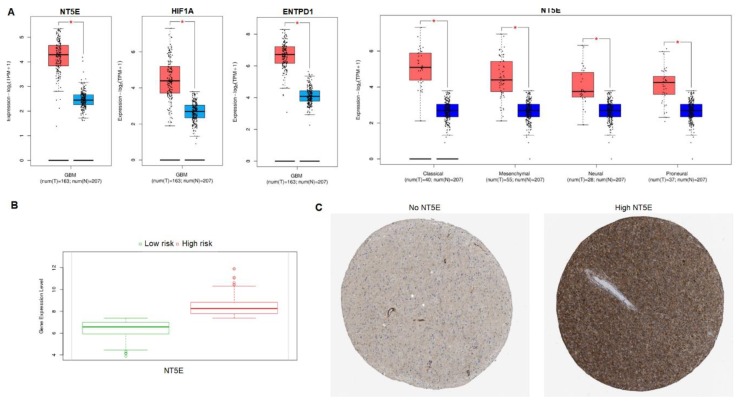
Expression of *NT5E* in glioblastoma (GBM). (**A**) Expression of *NT5E*, *HIF1A*, and *ENTPD1* in GBM (red) and normal brain (blue) based on TCGA data analyzed by GEPIA2 (*n* = 163). *NT5E* expression was also analyzed with TCGA data stratified into GBM subtypes: Classical, mesenchymal, neural, and proneural. All samples showed higher target gene expression in GBM compared to normal tissue (* *p* < 0.01). (**B**) Expression of *NT5E* in GBM based on risk group. High risk patients showed higher expression of *NT5E* compared to low risk patients. Risk group analysis was done in SurvExpress (*p* = 4.91 × 10^−6^; *n* = 538). (**C**) Immunohistochemical staining of glioma tissue obtained from the Human Protein Atlas showing *NT5E*-negative tissue (female, age 36; **left**) and high *NT5E*-expressing glioma tissue (male, age 71; **right**).

We first analyzed the expression of *NT5E*, the gene encoding the ectoenzyme CD73, in tissues of GBM patients based on analysis of TCGA RNAseq data for 163 patients using GEPIA2. Significant expression of *NT5E* was observed in GBM tumors compared to normal tissue (normal brain tissue data from TCGA and GTEx; Figure 1A). The *p*-value was set to 0.01. When further stratified by molecular GBM subtype—classical, mesenchymal, neural, and proneural—we found that expression of *NT5E* was also elevated in all subtypes compared to normal brain tissue (Figure 1A). Stratification into GBM subtypes was available for 40 classical, 55 mesenchymal, 28 neural and 37 proneural GBM samples. 

Because hypoxia-inducible factor 1α (HIF-1α) is considered a key regulator of the responses of cells to hypoxia with a role in driving the expression of CD73, we sought to determine whether the expression of *HIF1A*, the gene encoding the HIF-1α protein, was also upregulated in GBM. Our analysis matched the observation we had made for *NT5E*, in that GBM displayed significantly elevated expression of *HIF1A* compared to that in non-diseased brain tissue (Figure 1A). As a second enzyme of the adenosinergic cascade working in tandem with CD73, CD39 expression is essential to adenosine production as it catalyzes the first step in the ATP → AMP → adenosine pathway. Gene expression analysis confirmed that *ENTPD1*, which encodes CD39, was also significantly upregulated in GBM compared to normal tissue (Figure 1A). We then analyzed the expression of the four adenosine receptors: *ADORA1*, *ADORA2A*, *ADORA2B*, and *ADORA3* in GBM (Figure S1). Among the four adenosine receptors, only expression of *ADORA3* (which encodes the adenosine A_3_ receptor and is expressed on NK cells) was significantly upregulated in GBM compared to normal tissue (Figure S1). *ADORA3* expression was significantly upregulated in each of the four GBM subtypes as well. When stratified by risk groups, high-risk patients displayed significantly more *NT5E* compared to low risk patients (*p* = 4.91 × 10^−6^; Figure 1B). TCGA Wanderer analysis further confirmed the upregulated expression of *ADORA3* in GBM, however its reliance on only 5 normal brain samples places limited weight on this data. Immunohistochemical staining of glioma tissue obtained from the Human Protein Atlas shows distinct staining of *NT5E*-negative tissue (female, age 36; Figure 1C, left) compared to highly *NT5E*-expressing glioma tissue (male, age 71; Figure 1C, right).

### 3.2. The Role of nt5e as Prognostic Factor in GBM Survival

**Figure 2:**
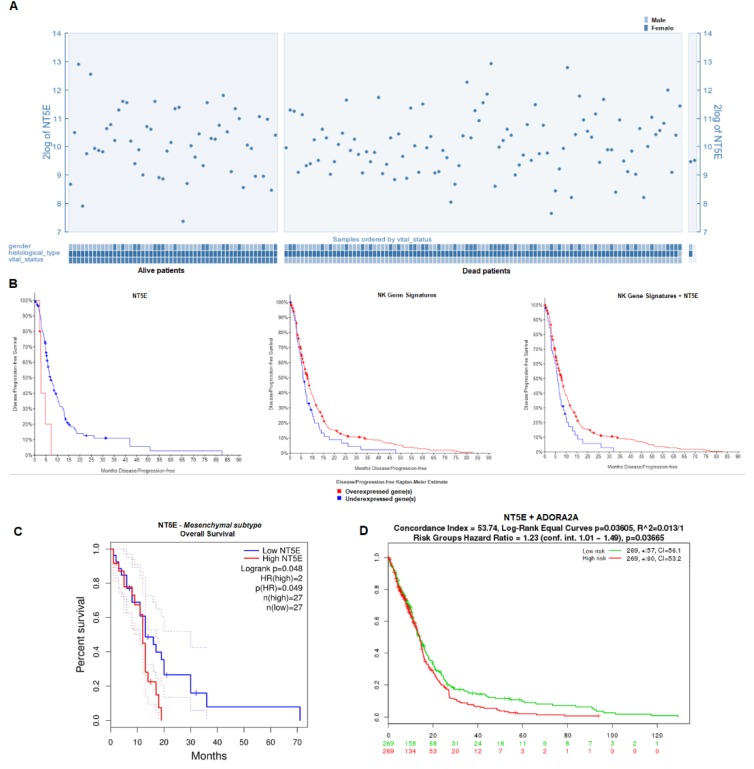
Survival analysis in the context of *NT5E* and natural killer (NK) gene signature expression. (**A**) Expression of *NT5E* in GBM patient samples plotted on the basis of patient vital status. Analysis was done in R2 (*n* = 540). (**B**) Disease-free survival of GBM patients on the basis of *NT5E* expression level from TCGA RNASeq V2 RSEM data (*p* = 0.0039; z = 2; **left**; *n* = 166); NK gene signatures comprising 13 NK-specific genes from U133 Affymetrix gene expression data (*p* = 0.0285; **middle panel**); and both *NT5E* and NK gene signatures from U133 Affymetrix gene expression data (*p* = 0.0109; **right**). Kaplan–Meier plots were generated in cBioPortal (*n* = 533). (**C**) Overall survival of GBM patients with the mesenchymal subtype based on *NT5E* expression. Analysis was done in GEPIA2 (*n* = 163). (**D**) Overall survival stratified by risk group for patients expressing *NT5E* and *ADORA2A*. Analysis was done in R2.

Correlation between gene expression and survival was next performed for both individually-queried genes and gene signatures. Analysis of *NT5E* expression based on TCGA RNASeq RSEM data for 153 patients showed no correlation to vital status when stratified by mortality of GBM patients in R2 (*p* = 0.654; Figure 2A). Kaplan–Meier survival plots were generated in cBioPortal and GEPIA2 based on a 50% median expression cutoff for high- and low-expressing groups. The Cox proportional hazards model was used to determine the hazards ratio for each survival plot. Glioblastoma patients who had downregulated CD73 expression recorded a prolonged median disease-free survival of 7.62 months, whereas patients who had upregulated CD73 had a disease-free survival of 4.73 months (*p* = 0.0039; z = 2; Figure 2B, left). While a longer OS was also recorded (upregulated CD73 = 14.52 months vs. downregulated CD73 = 14.06 months), this was not statistically significant (*p* = 0.667). OS findings in relation to *NT5E* expression were confirmed in a separate analysis based on the TCGA datasets by GEPIA2 (data not shown). No significant correlation with survival was observed for either *ENTPD1* or *HIF1A* gene expression (data not shown). However, statistically-significant lower median disease-free survival was observed for cases where both *NT5E* and *ENTPD1* were upregulated (5.16 months vs. 7.62 months; *p* = 0.0143) (Figure S2).

The lack of correlation between *NT5E* expression and OS was maintained when the analysis was stratified into three GBM subtypes: Neither classical, proneural, nor neural GBM samples showed any significant correlation between *NT5E* expression and survival. Interestingly, we observed significant negative correlation between OS and expression of *NT5E* for the mesenchymal GBM subtype (*p* = 0.048; Figure 2C). A low (<50%) expression of *NT5E* resulted in statistically-significant prolonged OS for this GBM subtype analyzed in GEPIA2. Also of note is the fact that expression of *ENTPD1* did not, unlike *NT5E*, correlate with survival in any of the GBM subtypes analyzed, including mesenchymal (data not shown). 

Having observed this correlation, we sought to determine the effect of gene signatures corresponding to NK cells in the analyzed samples on survival data. The gene signature group consisted of the genes *XCL2*, *PRF1*, *KLRF1*, *KLRD1*, *KLRC3*, *KLRC1*, *IL2RB*, *IL18RAP*, *GNLY*, *CST7*, *CHST12*, *CD244*, and *CD160* [23]. Survival analysis based on U133 mRNA microarray TCGA data (z = 1) revealed a significantly higher median disease-free survival (7.85 months vs. 5.81 months, *p* = 0.0285; Figure 2B, middle panel) for cases with the NK gene signatures over-expressed compared to cases for which NK-specific genes were under-expressed. Survival analysis on the basis of the expression of NK-specific genes alongside *NT5E* showed that even in samples with high *NT5E* expression, median disease-free survival in the presence of over-expressed NK signatures was higher than those cases with low NK and *NT5E* genes (7.82 months vs. 5.81 months, z = 1; *p* = 0.0109; Figure 2B, right). Moreover, high-risk patients co-expressing both *NT5E* and the adenosine A_2A_ receptor (*ADORA2A*) reported a higher OS compared to low-risk patients, based on analysis by SurvExpress (*p* = 0.0366; Figure 2D).

### 3.3. CD73 Gene Expression Based on Length of Patient Survival and Tumor Location

Analysis of gene expression data from patients extracted from GSE53733 showed that expression of *NT5E* did not correlate with length of patient survival. Long-term survivors (>36 months OS), short-term survivors (<12 months OS), and intermediate OS patients had similar expression levels of *NT5E* (Figure S3). Similarly, tumor location did not show to affect expression of *NT5E* based on gene expression data from GSE36245, with cerebellar, frontal, temporal, occipital, and parietal tumors all showing comparable levels of *NT5E* expression (Figure S4).

### 3.4. Correlation in Gene Expression Pairs

**Figure 3:**
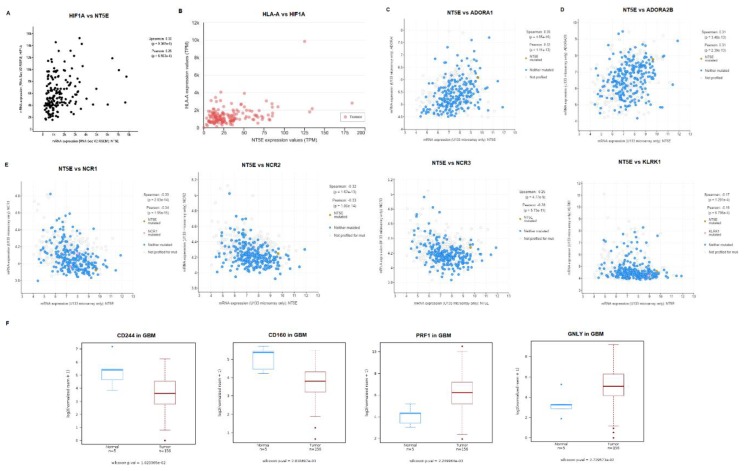
Correlation of expression of *NT5E* and NK gene signatures in GBM. (**A**) Co-expression of *NT5E* and *HIF1A* in GBM. Data were analyzed in cBioPortal using U133 Affymetrix microarray data (z = 1; *n* = 533). (**B**) Co-expression of *NT5E* and *HLA-A* based on TCGA data in UALCAN (*n* = 156). (**C**) Co-expression of *NT5E* and *ADORA1* in GBM using the TCGA database analyzed in cBioPortal on the basis of U133 Affymetrix microarray data (z = 1; *n* = 533). (**D**) Co-expression of *NT5E* and *ADORA2B* in GBM using the TCGA database analyzed in cBioPortal on the basis of U133 Affymetrix microarray data (z = 1). (**E**) Co-expression of *NT5E* and genes representing NK activating receptors: *NCR1*, *NCR2*, *NCR3*, and *KLRK1* in GBM. Data were analyzed in cBioPortal using U133 Affymetrix microarray data (z = 1). (**F**) Box-plots of the expression of genes associated with NK cells in GBM (red) and normal tissue (blue): *CD244*, *CD160*, *PRF1* and *GNLY* based on TCGA data analyzed in TCGA Wanderer (*n* = 156).

Correlation analysis of gene pairs for TCGA GBM data were carried out to determine gene pairs which show co-expression in GBM. A positive correlation was observed for the expression of *NT5E* and *HIF1A* genes (Spearman’s rank correlation coefficient, ρ = 0.3; *p* = 9.302 × 10^−5^; Figure 3A). Analysis of GBM gene expression by UALCAN revealed *HLA-A*, which encodes human leukocyte antigen A, to be among the most positively-correlated genes expressed alongside *NT5E* (Pearson’s rank correlation coefficient = 0.39; Figure 3B). 

Correlation between the expression of *NT5E* and genes encoding the four adenosine receptors *ADORA1, ADORA2A, ADORA2B*, and *ADORA3* revealed significant positive correlation between *NT5E* and *ADORA1* (ρ = 0.345, *p* = 4.85 × 10^−16^, Figure 3C) and *ADORA2B* (ρ = 0.311, *p* = 3.48 × 10^−13^, Figure 3D). Weak correlation was observed between the expression of *NT5E* and *ADORA3* (ρ = 0.180, *p* = 3.68 × 10^−5^, Figure S4B). No correlation was observed between the expression of *NT5E* and *ADORA2A* (ρ = −0.05, *p* = 0.217, Figure S4A).

We also analyzed the correlation in expression of *HIF1A* and the four adenosine receptor-encoding genes. We observed significant positive correlation (ρ = 0.31, *p* = 2.65 × 10^−4^) between the expression of *ADORA3* and *HIF1A* in GBM samples (Figure S5D). Low positive correlation was noted for the co-expression of *HIF1A* and *ADORA1* (ρ = 0.26, *p* = 1.925 × 10^−3^; Figure S5A) and *HIF1A* and *ADORA2B* (ρ = 0.22, *p* = 1.01 × 10^−2^; Figure S5C), while no correlation was shown between *HIF1A* and *ADORA2A* (ρ = 0.06, *p* = 0.521; Figure S5B). Interestingly, *ADORA3* was also the only receptor that was upregulated in GBM compared to normal brain (Figure S1).

We also carried out correlation analysis between expression of *NT5E* and individual genes representing NK cell activation markers. Interestingly, a significant negative correlation was observed with the co-expression of *NT5E*/*NCR1* (ρ = −0.33; *p* = 2.03 × 10^−14^; Figure 3E), and *NT5E*/*NCR2* (ρ = −0.32; *p* = 1.62 × 10^−13^; Figure 3E). Low negative correlation was observed for expression of gene pairs *NT5E*/*KLRK1* (ρ = −0.17; *p* = 1.29 × 10^−4^; Figure 3E), while a weak negative correlation was observed with the co-expression of *NT5E*/*NCR3* (ρ = −0.25; *p* = 4.77 × 10^−9^; Figure 3E). TCGA Wanderer analysis revealed that among the 13 NK gene signatures surveyed, expression of CD244 (*p* = 0.01), CD160 (*p* = 0.00281), PRF1 (*p* = 0.00225), and GNLY (*p* = 0.0273) was higher in GBM compared to normal tissue (Figure 3F).

### 3.5. Tumor-Infiltrating Natural Killer Cells in GBM

**Figure 4:**
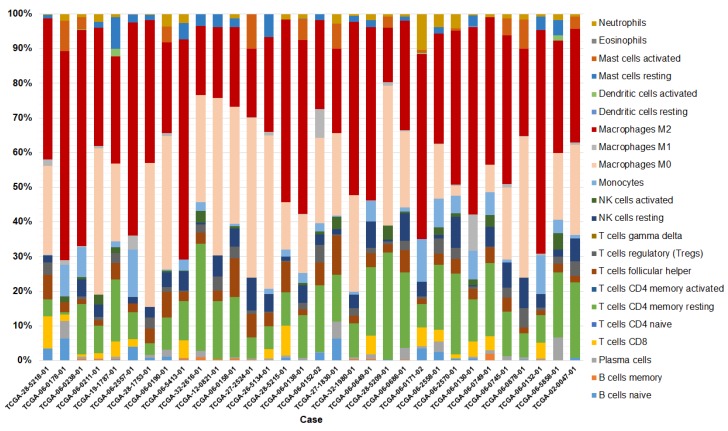
Profiling of infiltration of immune cells into GBM. Deconvoluted immune cell subsets from The Cancer Genome Atlas (TCGA) data by CIBERSORT indicate NK cells represent up to ~9.48% of GBM immune infiltrates. Data have been trimmed to include cases for which gene expression was *p* < 0.05.

To determine the proportion of immune cell subpopulations, particularly NK cells, represented in the gene signatures associated with GBM datasets from TCGA, we extracted RNAseq V2 RSEM data and used CIBERSORT to deconvolute gene expression data into immune cell subsets. Deconvolution was done based on the CIBERSORT leukocyte signature gene matrix (LM22). We filtered out samples with low significance to retain *p* < 0.05. Deconvolution of immune cell subsets identified in GBM samples revealed the presence of 19 immune cell subpopulations within the GBM TCGA gene matrix-all cell types except γδ T cells, naïve CD4^+^ T cells, and activated memory CD4^+^ T cells were identified (Figure 4). Most abundant were M2-polarized macrophages (39.24%), followed by uncommitted (M0; 21.04%) macrophages. The next most abundant subgroup of identified immune cells are CD4^+^ memory resting T cells (13.83%). The least abundant cell populations deconvoluted from gene expression data are eosinophils, plasma cells, and dendritic cells. Activated NK cells represented 1.07% of the total immune cell population in the analyzed GBM samples, while resting NK cells represented 4.09% of all immune cells. In comparison, CD8^+^ T cells represented 2.02% of the total immune cell population. 

**Figure 5:**
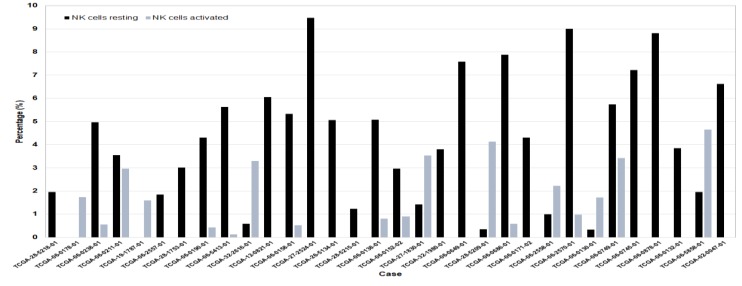
Proportion of NK cells identified in GBM TCGA samples. Deconvoluted RNASeq transcriptional data into resting and activated NK cells plotted as a percentage of these cells’ presence in individual TCGA samples for which *p* < 0.05.

An analysis of NK cells quantified in each of the individual GBM cases for which *p* < 0.05, reveals that NK presence accounts for between 0 and 4.66% for activated NK cells, and between 0 and 9.48% for resting NK cells in GBM (Figure 5).

Overall, our analysis on the presence of NK cells in GBM based on gene expression data shows that there might be positive prognostic significance in enhanced presence of NK cells in this tumor. While CIBERSORT data show that NK cells account for under 10% of the immune subsets in GBM, this analysis does not provide clinical outcome information. In other words, the data indicate that among all GBM cases recorded in the TCGA database (independent of survival), NK cell presence accounts for a very small fraction of all immune populations. However, when data is stratified by patient outcome, as discussed in Section 3.2, the gene signature corresponding to NK cells indicates that there could be a positive effect on disease-free survival when NK cell genes are over-expressed. Collectively, while this data dos not provide an indication of the abundance of NK cells required for improved patient outcome, it does suggest that there is likely to be a clinical benefit in patients bearing CD73^+^ tumors that show higher infiltration of NK cells.

## 4. Discussion

GBM is an uncurable tumor associated with a profoundly immunosuppressive pathology and characterized by upregulation of the ectoenzyme CD73, the product of the *NT5E* gene. CD73 is one half of a bi-enzymatic cascade: in association with CD39, it catalyzes the conversion of extracellular ATP into AMP and, ultimately, adenosine [24]. Adenosine signaling on tumor-infiltrating NK cells drives immunosuppression of NK cell effector responses and metabolic functions by binding to one or more of four G protein-coupled adenosine receptors-A_1_, A_2A_, A_2B_, and A_3_ [14,25]. It is known that CD73 is overexpressed in glioblastoma multiforme [26], where it contributes to its diverse pathologies. Within the context of GBM, CD73 was shown to contribute to the drug-resistance phenotype characteristic of GBM. Knocking down CD73 expression was, for instance, shown in a recent study to sensitize GBM cells to treatment by the drug vincristine. This was associated with downregulation of expression of the multiple drug associated protein 1 (Mrp1) [27]. Glioma-associated CD73 was also shown to drive adenosinergic immunosuppression in concert with CD39 present on infiltrating CD4^+^CD39^+^ T lymphocytes [28]. CD73 has also emerged as regulator of the invasive phenotype of GBM [29] by mediating glioma cell adhesion and tumor cell-extracellular matrix interactions [30]. The role of adenosine receptors in GBM has also received considerable attention. The CD73-A_2B_ axis was described, in a recent study, as playing a significant role in promoting GBM growth, invasiveness and angiogenesis [31], while Torres et al. [10] showed that extracellular adenosine signals on glioblastoma stem-like cells via the A_3_ receptor to promote their migration and invasion. Despite its recognized roles in the pathogenesis of GBM, the relationship between CD73 and patient survival is less known. Moreover, we and others have shown that adenosinergic signaling is an important set of immunosuppressive signaling mechanisms which directly impair NK cell cytotoxicity [9]. However, the relationship between CD73 expression and NK presence in GBM remains underexplored.

In the current study, we systematically analyzed *NT5E* expression in GBM by utilizing in silico online expression databases and bioinformatics data mining tools to determine the relationship between *NT5E* expression, patient survival and NK cell presence in GBM. Analysis of datasets revealed that *NT5E* expression is significantly augmented in GBM compared to normal brain tissue. Expression of *NT5E* is elevated in each of four main GBM subsets, namely classical, mesenchymal, proneural, and neural, compared to normal brain. Our analysis indicated that GBM patients with overexpressed *NT5E* had a shorter disease-free survival compared to patients with under-expressed *NT5E*. This is in agreement with previous analyses [28]. GBM patients characterized by the mesenchymal GBM subtype also had longer OS when *NT5E* was under-expressed. The mesenchymal subtype is associated with the worst prognosis in GBM patients by driving aggressiveness and treatment resistance [32]. As a result, there is currently no successful treatment option available against the mesenchymal phenotype. The poor survival associated with *NT5E* expression in this subtype might imply pathogenesis associated with immune dysfunction due to immunosuppressive adenosinergic signaling to be contributing to the malignancy of mesenchymal GBM. Furthermore, the overexpression of *ADORA3* in GBM observed here corroborates the important role of the A3 receptor, reported in other studies, in driving GBM progression [33,34]. Interestingly, the higher OS for patients co-expressing *NT5E* and *ADORA2A* might be indicative of a higher immune cell infiltrate, given that other immune cells alongside NK cells express *ADORA2A*. The higher presence of immune effectors might counteract negative adenosinergic signaling induced by the *NT5E*-*ADORA2A* axis. The A_2B_ receptor, encoded by *ADORA2B*, has low affinity for adenosine though a wide tissue expression. It has recently been implicated in control of GBM stem cell survival, alongside *ADORA1* [35]. The correlation in expression of *NT5E* and *ADORA2B* could be related to its wider presence on non-immune cells including GBM itself, which might explain, at least partly, the lack of any correlation to patient outcome. However, these findings warrant further investigation. On the other hand, the A_1_ receptor has been associated with a pro-inflammatory response [9]. Its co-expression with *NT5E* on GBM could be related to its role in cancer pathology, emerging from a number of recent studies [35,36]. It bears mentioning that further validation of these findings is necessary to establish the role of adenosine receptors in GBM. 

Significantly positive correlation of expression between *NT5E* and *HLA-A*, which encodes the human leukocyte antigen class I A, a ligand for killer inhibitory receptors KIR2DL1, KIR3DL2, and KIR3DL3 [37,38] expressed on NK cells, is indicative of a potential contribution to failed “missing self” recognition of GBM by NK cells due to CD73. Our analysis showed that the expression of *ADORA1* and *ADORA2B* correlated to that of *NT5E*. This might be in line with a recent report which indicated that the A_2B_ receptor plays a role in promoting the progression of GBM [31]. Correlative analysis also showed that expression of *ADORA2A*, which encodes the A_2A_ receptor on NK cells, correlated with *NT5E* expression in GBM to induce shorter OS in high-risk patients. Collectively, these findings suggest that CD73 might have a direct effect on specific anti-tumor functions of NK cells by promoting escape from immune recognition and effector functions. It bears mentioning that *ADORA2A* expression is not limited to NK cells, so our observations do not exclude the potential involvement of cells other than NK cells in these outcomes.

Though NK cells have been found in GBM, the number of NK cells infiltrating GBM is low [12,13], and most of the GBM-infiltrating NK cells are non-functional [39]. Despite the demonstrated attractiveness of using NK cells as immunotherapies against GBM, little is still known about their presence and role in GBM. We found that mRNA signatures corresponding to 13 genes expressed by NK cells [23] correlated to improved median disease-free survival of GBM patients. This was also the case when co-expressed with *NT5E*, suggesting that infiltration of NK cells might provide protection over the pathophysiology due to CD73. Significant negative correlation between the expression of *NT5E* and individual genes *NCR1* and *NCR2*, which encode the activating receptors NKp46 and NKp44, respectively, suggests that CD73 activity in GBM might impair the activation of NK cells by downregulating individual receptor-specific functions. While our findings suggest that NK cells might induce a higher disease-free survival in GBM patients, correlation between the expression of *ADORA2A* and *NT5E* might infer immunosuppression due to adenosinergic signaling on immune cells including NK as well as other cells, leading to poorer overall survival and suggesting that a complex interplay of tumor microenvironment factors is likely to affect pathology of GBM.

In concordance with previous reports, infiltration of NK cells indicated that these cells represent the minority of immune infiltrates in GBM. It is interesting to point out that other effector cells, such as CD8^+^ T cells, were similarly represented. A recent report indicated that GBM promotes polarization of macrophages to the M2 phenotype [34]. Indeed, our analysis showed that the most abundant cell type in GBM are M2 macrophages, followed by M0 macrophages. A distinct proportion of resting NK cells, more abundant in quantity than activated NK cells, was also recorded. Typically, resting NK cells represent a functionally- and phenotypically-distinct NK cell subset characterized by lower expression of activating NK receptors including NKG2D and NKp46 [40,41]. Though functional, these cells are less cytotoxic against cancer targets than activated NK cells [42]. However, while various groups have reported that NK cells are able to kill GBM cells and GBM-like stem cells, this is not the case for resting NK cells [43]. NK cells deconvoluted from transcriptional data in our analysis could represent functionally exhausted NK cells that have infiltrated GBM. These NK cells are likely to have impaired protein expression signatures, thus helping explain the negative correlations between individual activating NK genes and *NT5E* expression observed in our analysis. Interestingly, correlation between elevated expression of genes encoding certain specific NK receptors in GBM compared to normal tissue is suggestive of the fact that NK cells might be responding to CD73 signaling via very specific functional mechanisms and activation patterns.

Taken together, our findings suggest that CD73 is a potentially significant prognostic biomarker for GBM, particularly the mesenchymal subset of GBM. Moreover, expression of CD73 correlates negatively with the expression of activating NK receptors, although the presence of NK cells in GBM indicates improved disease-free survival. Overall, this is the first study to actively survey public gene expression data to establish the role and relationship between CD73 and NK cells in GBM and suggest therapeutic value owing to their intratumoral presence.

## 5. Conclusions

As an uncurable disease, GBM remains elusive, and the heavy immunosuppression associated with its pathology is of considerable interest for the development of new immunotherapeutic approaches. This analysis reveals CD73 to be a significant target in GBM and suggests there is therapeutic value in augmenting NK cell presence and function in GBM. Because CD73 is an enzymatically active cancer-associated antigen, alleviating the CD73-driven adenosinergic immunosuppression could be mechanisms by which immune cell infiltration into GBM could be augmented. Moreover, our findings infer a potential role of CD73 in driving the activation, or rather exhaustion, of NK cells in GBM. These findings contribute to our understanding of the role of adenosine signaling and NK cell function in GBM and pave the way for approaches that can restore immune cell function to more effectively target GBM.

## Supplementary Materials

The following are available online at https://www.mdpi.com/2077-0383/8/10/1526/s1, Figure S1: Expression of *ADORA1, ADORA2A, ADORAB* and *ADORA3* in GBM and normal tissue, Figure S2: Survival analysis based on expression of *NT5E* and ENTPD1 in GBM, Figure S3: Expression of *NT5E* in GBM based on tumor survival time and tumor location, Figure S4: Correlation of expression of *NT5E* and genes expressing adenosine receptors A2A (*ADORA2A*), and A3 (*ADORA3*), Figure S5: Correlation of expression of *HIF1A* and genes expressing adenosine receptors A1 (*ADORA1*), A2A (*ADORA2A*) and A2B (*ADORA2B*), Figure S6: Data analysis tools used in this work.
