# Supplementary material for: NT5E/CD73 as Correlative Factor of Patient Survival and Natural Killer Cell Infiltration in Glioblastoma
Source: J Clin Med. 2019 Sep 23;8(10):1526. doi: 10.3390/jcm8101526 (PMC6832588; doi:10.3390/jcm8101526)
Supplement: Supplementary file 1 [file jcm-08-01526-s001.zip › jcm-593303-SI.pdf]

Journal of
Clinical Medicine

MDPI

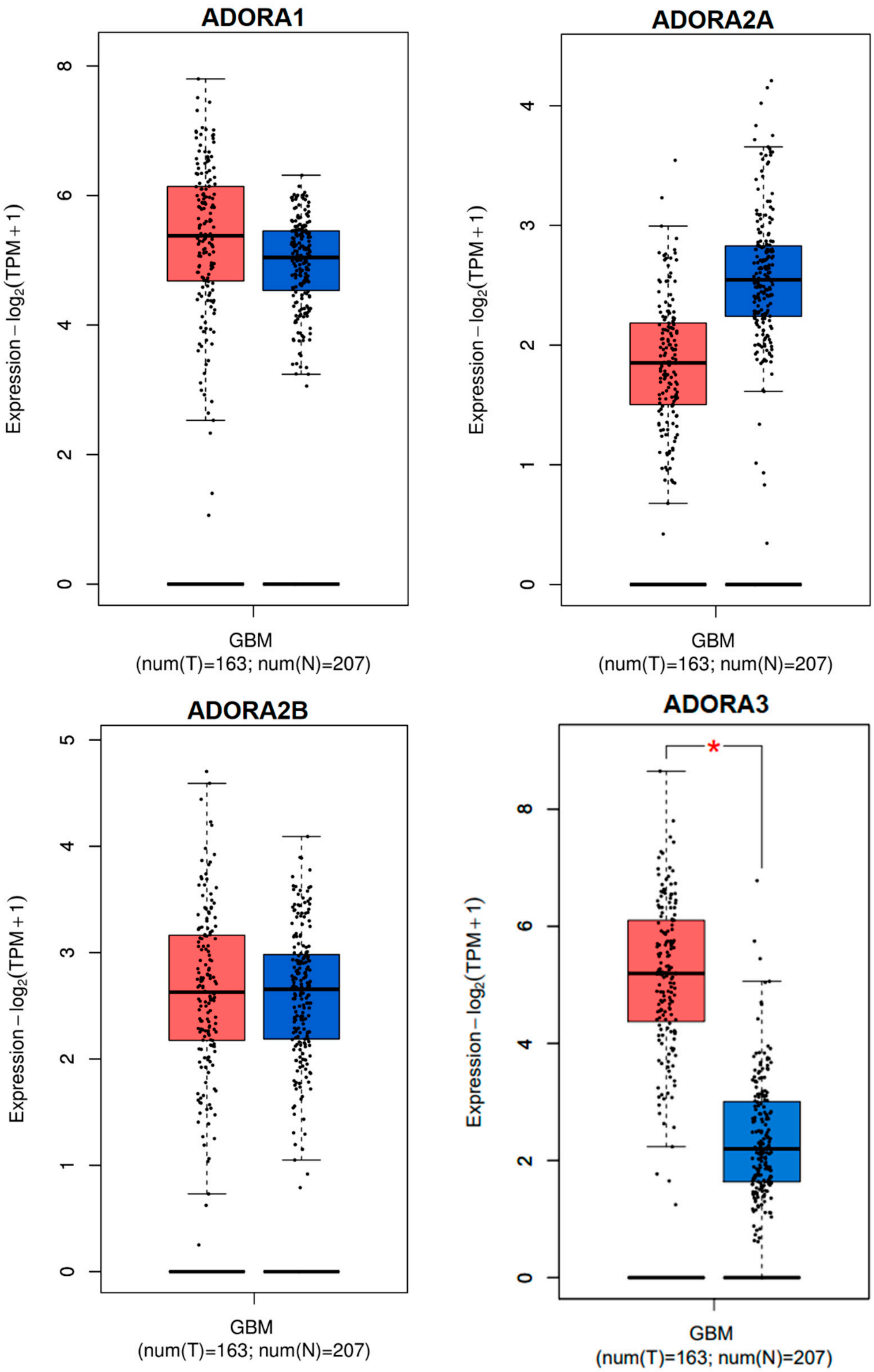

Figure S1. Expression of *ADORA1*, *ADORA2A*, *ADORA2B* and *ADORA3* in GBM and normal tissue. *ADORA3* expression was significantly upregulated in GBM (red) compared to normal brain tissue (blue), based on TCGA data analyzed by GEPIA2 (* $p < 0.01$).

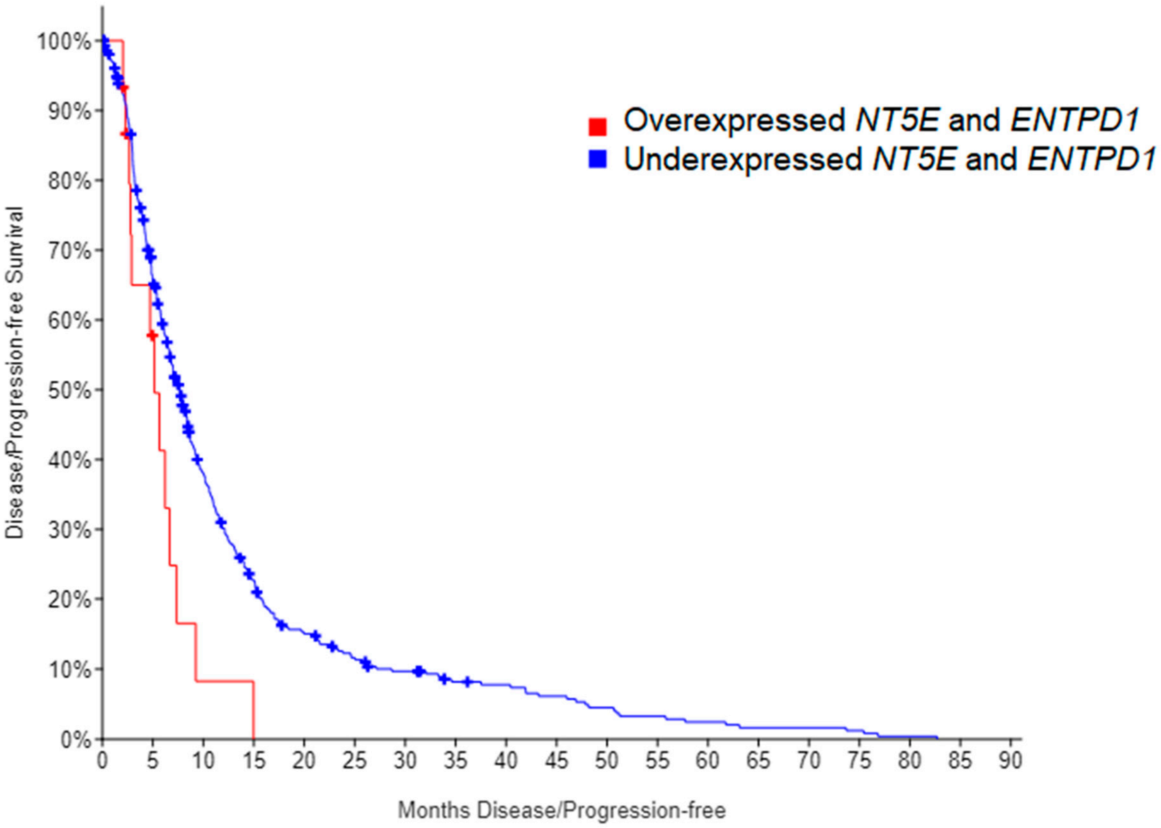

Figure S2. Survival analysis based on expression of *NT5E* and ENTPD1 in GBM. (**A**) Kaplan-Meier curve of disease-free survival of patients based on the expression of *NT5E* and *ENTPD1* in GBM. Patients with both genes overexpressed (red, 5.62 months) showed higher median disease-free survival compared to patients with both genes underexpressed (blue, 7.62 months) based on RNASeq TCGA data analyzed by cBioPortal (z = 2; $p = 0.0143$).

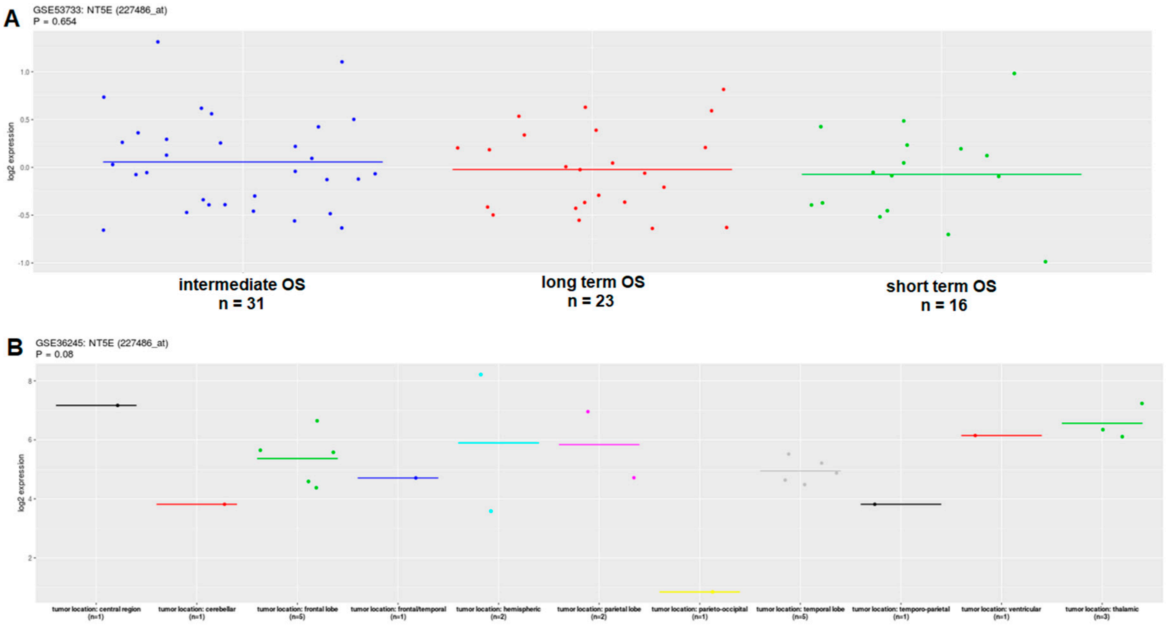

Figure S3. Expression of *NT5E* in GBM based on tumor survival time and tumor location. (**A**) Analysis of *NT5E* expression within the GSE53733 dataset stratified by length of patient survival (p = 0.654). (**B**) Expression of NT5E in GBM within the GSE36245 dataset based on tumor location (p = 0.08). Data wereas analyzed using shinyGEO.

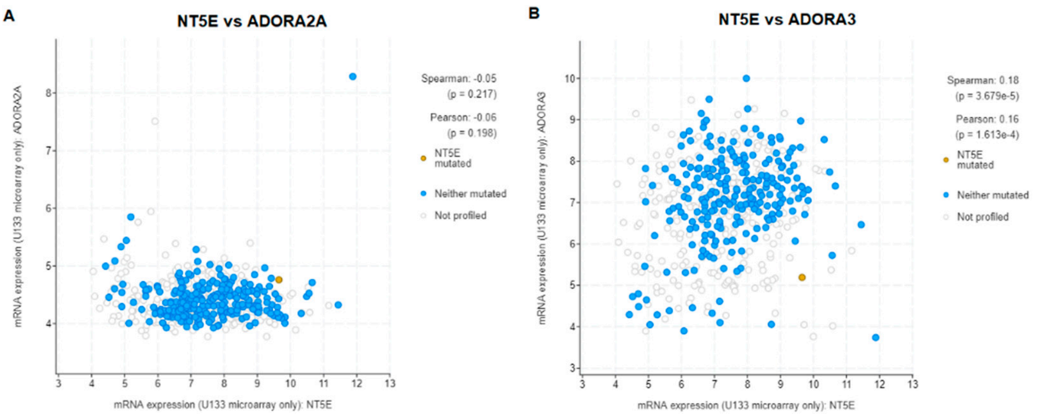

Figure S4. Correlation of expression of *NT5E* and genes expressing adenosine receptors A2A (*ADORA2A*), and A3 (*ADORA3*). (**A**) Co-expression of *NT5E* and *ADORA2A* in GBM. (**B**) Co-expression of *NT5E* and *ADORA3* in GBM. Data wereas analyzed using the TCGA database analyzed in cBioPortal on the basis of U133 Affymetrix microarray data (z = 1).

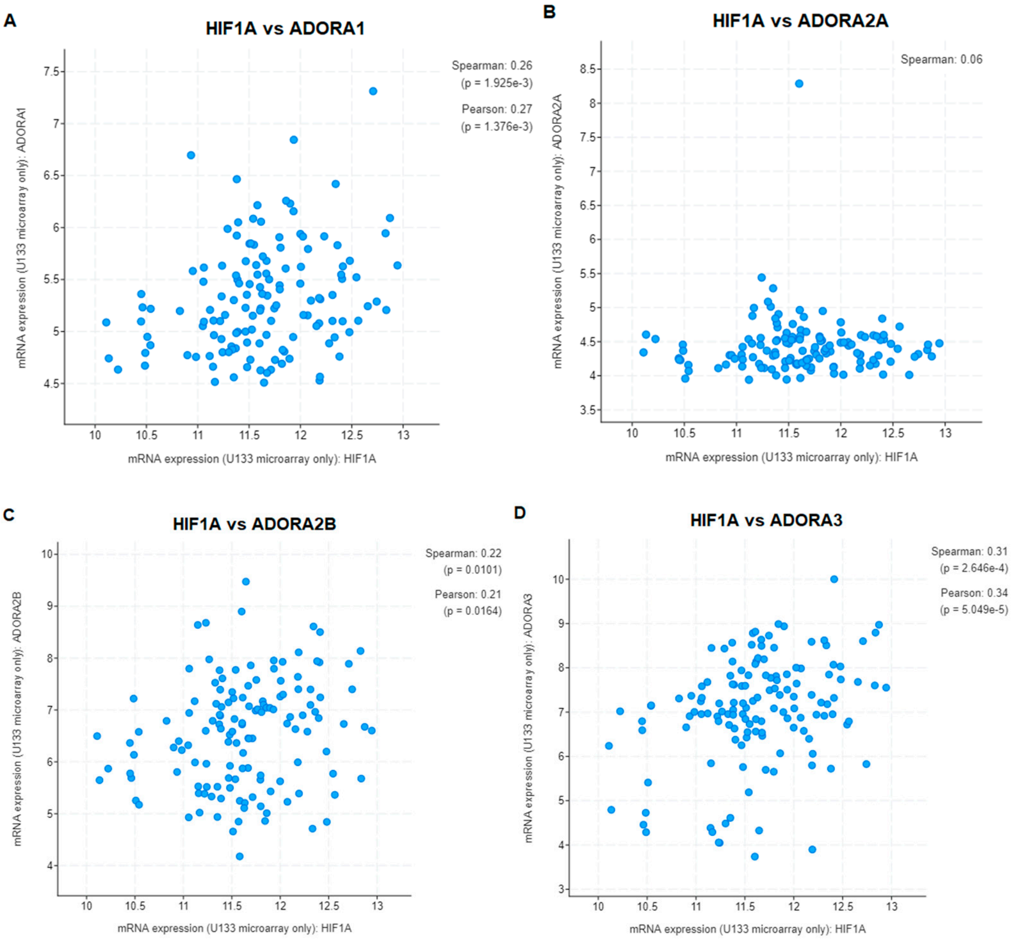

Figure S5. Correlation of expression of *HIF1A* and genes expressing adenosine receptors A1 (*ADORA1*), A2A (*ADORA2A*) and A2B (*ADORA2B*). (**A**) Co-expression of *HIF1A* and *ADORA1* in GBM. (**B**) Co-expression of *HIF1A* and *ADORA2A* in GBM. (**C**) Co-expression of *HIF1A* and *ADORA2B* in GBM. (**D**) Co-expression of *HIF1A* and *ADORA3* in GBM. Data were~~as~~ analyzed using the TCGA database analyzed in cBioPortal on the basis of U133 Affymetrix microarray data (z = 1).

**Dataset analysis tool**	**Number of GBM cases**	**Analysis type**
GEPIA2	163 (207 GTEx normal brains)	Gene expression *vs* normal brain (GTEx), correlation, survival
TCGA Wanderer	156 (5 TCGA non-GBM tissues)	Gene expression *vs* non-GBM tissue (TCGA)
R2	540	Gene expression by vital status
SurvExpress	538	Gene expression by risk group, survival
cBioPortal	166 (RNASeq V2), 533 (U133 microarray)	Correlation, survival
UALCAN	156 (5 TCGA non-GBM tissues)	Gene expression *vs* non-GBM tissue (TCGA), correlation

Figure S6. Data analysis tools used in this work. GBM cases correspond to the number of TCGA datasets utilized for analysis by each database tool. TCGA's tissue normal samples represent tissue from individuals with GBM, while GTEx normal tissue samples represent brain tissue from healthy individuals.
